# Molecular Analysis Reveals a High Diversity of Anopheline Mosquitoes in Yanomami Lands and the Pantanal Region of Brazil

**DOI:** 10.3390/genes12121995

**Published:** 2021-12-16

**Authors:** Teresa Fernandes Silva-do-Nascimento, Jordi Sánchez-Ribas, Tatiane M. P. Oliveira, Brian Patrick Bourke, Joseli Oliveira-Ferreira, Maria Goreti Rosa-Freitas, Ricardo Lourenço-de-Oliveira, Mariana Marinho-e-Silva, Maycon Sebastião Alberto Santos Neves, Jan E. Conn, Maria Anice Mureb Sallum

**Affiliations:** 1 Laboratório de Mosquitos Transmissores de Hematozoários, Instituto Oswaldo Cruz, Fundação Oswaldo Cruz, Rio de Janeiro 21040-360, Brazil; teresa.nascimento@ioc.fiocruz.br (T.F.S.-d.-N.); elsanchez@gmail.com (J.S.-R.); lila@ioc.fiocruz.br (J.O.-F.); goreti.freitas@geniac-academy.com (M.G.R.-F.); lourenco@ioc.fiocruz.br (R.L.-d.-O.); mariana.marinho@inpi.gov.br (M.M.-e.-S.); mayconsn@ioc.fiocruz.br (M.S.A.S.N.); 2 Laboratório de Imunoparasitologia, Instituto Oswaldo Cruz, Fundação Oswaldo Cruz, Rio de Janeiro 21040-360, Brazil; 3 Distrito Sanitário Especial Indígena Yanomami, Roraima 69301-080, Brazil; 4 Departamento de Epidemiologia, Faculdade de Saúde Pública, Universidade de São Paulo, São Paulo 01246-904, Brazil; masallum@usp.br; 5 Walter Reed Biosystematics Unit, Museum Support Center MRC-534, Smithsonian Institution, 4210 Silver Hill Rd., Suitland, MD 20746, USA; bourkeb@si.edu; 6 Walter Reed Army Institute of Research, 503 Robert Grant Avenue, Silver Spring, MD 20910, USA; 7 Department of Entomology, Smithsonian Institution—National Museum of Natural History, 10th St. NE & Constitution Ave. NE, Washington, DC 20002, USA; 8 Geniac Ltd., São Paulo 01031-902, Brazil; 9 Instituto Nacional da Propriedade Industrial, Rio de Janeiro 20090-910, Brazil; 10 Wadsworth Center, New York State Department of Health, Albany, NY 12159, USA; jan.conn@health.ny.gov; 11 Department of Biomedical Sciences, School of Public Health, State University of New York, Albany, NY 12222, USA

**Keywords:** Anophelinae, diversity, mosquito, Yanomami, Pantanal

## Abstract

Identifying the species of the subfamily Anophelinae that are *Plasmodium* vectors is important to vector and malaria control. Despite the increase in cases, vector mosquitoes remain poorly known in Brazilian indigenous communities. This study explores Anophelinae mosquito diversity in the following areas: (1) a Yanomami reserve in the northwestern Amazon Brazil biome and (2) the Pantanal biome in southwestern Brazil. This is carried out by analyzing cytochrome c oxidase (*COI*) gene data using Refined Single Linkage (RESL), Assemble Species by Automatic Partitioning (ASAP), and tree-based multi-rate Poisson tree processes (mPTP) as species delimitation approaches. A total of 216 specimens collected from the Yanomami and Pantanal regions were sequenced and combined with 547 reference sequences for species delimitation analyses. The mPTP analysis for all sequences resulted in the delimitation of 45 species groups, while the ASAP analysis provided the partition of 48 groups. RESL analysis resulted in 63 operational taxonomic units (OTUs). This study expands our scant knowledge of anopheline species in the Yanomami and Pantanal regions. At least 18 species of Anophelinae mosquitoes were found in these study areas. Additional studies are now required to determine the species that transmit *Plasmodium* spp. in these regions.

## 1. Introduction

Despite many control and elimination efforts, malaria remains one of the most important public health problems globally and affects mainly developing countries [1]. In 2020, there were estimated 241 million cases of malaria worldwide and 620,000 deaths. Cases in the Americas, Brazil, Colombia, and Venezuela (Bolivarian Republic of) account for 77% of all cases in this region [2]. In Brazil, malaria cases increased by 3% from 2015 (138,004) to 2020 (142,124) [3]. The distribution of malaria cases within Brazil is highly heterogeneous, with the Amazon Region accounting for more than 99% of cases [4].

Malaria transmission dynamics in the Amazon is shaped by multiple factors, such as high temperature and humidity, mining activities, deforestation, human migration, road and hydroelectric plant construction, and human settlement near forested areas [5,6,7]. Changes to the anthropogenic and climatic environment can alter mosquito population size, habitat, and behavior, leading to increased *Plasmodium* transmission [8,9].

In Brazil, 54 species of mosquitoes among the *Nyssorhynhus*, *Kerteszia*, and *Anopheles* genera [10,11] are involved in *Plasmodium* transmission as primary or secondary vectors. *Anopheles* and *Nyssorhynchus* species are present throughout the Amazonian and the southeastern states, whereas *Kerteszia* species are associated with humid forests rich in bromeliads [12]. A recent study detected up to thirteen new species of mosquitoes in the Amazon region [13], demonstrating that anopheline diversity can be underestimated in poorly sampled areas.

The Yanomami people are the largest indigenous group in the Amazon. They inhabit an area of approximately 192,000 km^2^ between the extreme north Brazil (Amazonas and Roraima states) and southern Venezuela [14]. The open housing structure of settlements and geographic remoteness of Yanomami communities, taken together, result in persistent population exposure to mosquito bites and a major challenge for malaria control [15]. In addition, illegal land grabbing, mining, and logging increase the risk of acquiring malaria and create havoc for this highly vulnerable population [16]. Malaria is an increasing cause of morbidity among Yanomami communities [17], where multiple *Plasmodium* species circulate, with a predominance of low-density infections, especially among children, who are highly susceptible [18].

The Pantanal Brazilian Central Wetlands is one of the main extra-Amazon malaria-prone biomes in Brazil [19]. It is the smallest Brazilian biome but occupies almost 140,000 km^2^ of a seasonally flooded area in the Paraguay river basin, mostly in the states of Mato Grosso and Mato Grosso do Sul [20]. Despite the high biodiversity in this biome [21], few studies have explored Culicidae diversity there [22,23,24,25,26,27].

Among mosquito vectors of malaria protozoans in Brazil, many exist within cryptic species complexes, making correct species identification, based on morphology alone, difficult [28,29,30]. However, accurate species identification is essential for biodiversity studies [31], vector incrimination [32], and delineating interventions for malaria control focused on the species that disperse *Plasmodium* parasites [33].

Molecular tools are now routinely used to help resolve species identification in morphologically similar complexes, and the cytochrome c oxidase (*COI*) gene has become the marker of choice to establish molecular species barcodes [13,34,35,36]. Clearly, single locus approaches to species delimitation have limitations, with processes such introgression and incomplete lineage sorting potentially leading to shared genetic variation between closely related species [34,37]. However, the broad utility of the marker across species makes use of the *COI* barcode an important first step in species delimitation studies, which can be complemented by multi-gene, genomic, ecological, and morphological analyses. A range of species delimitation tools are now available to explore diversity within species complexes of Anophelinae, and the most popular of these for barcoding data can be divided into distance-based (Refined Single Linkage (RESL) [38], Automated Barcode Gap Discovery (ABGD) [39], Assemble Species by Automatic Partitioning (ASAP)) [40], phylogeny-based (Generalized Mixed Yule Coalescent (GMYC)) [41,42], and Poisson tree processes (PTP)) [43] approaches.

Little is known about the vector species and Anophelinae species complexes of the Pantanal [27] and the Yanomami territories [18,44,45]. A better understanding of species distribution, bionomic characteristics and vector status in these regions would provide important information for the development of more effective public health and malaria control strategies. The current study explored anopheline species diversity in these regions using *COI* gene data and a range of distance and phylogeny-based species delimitation tools.

## 2. Materials and Methods

### 2.1. Study Areas

**Figure 1:**
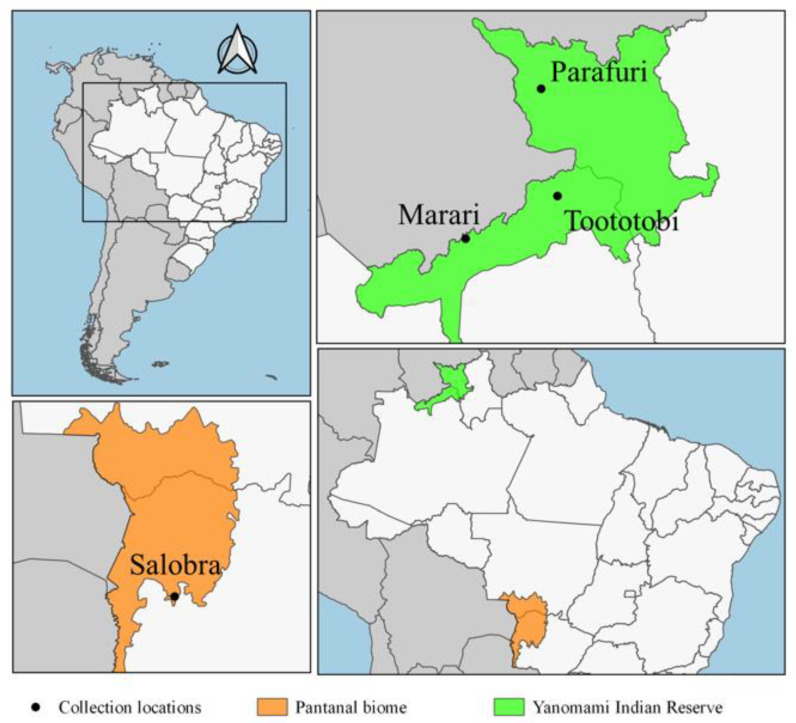
Specimen collection sites in Parafuri village (Roraima state), Toototobi and Marari villages (Amazonas state) in the Yanomami Indian Reserve, Brazilian Amazon, and Salobra (Mato Grosso do Sul state) in the Pantanal.

This study was conducted in four localities in Brazil: (1) Parafuri (3°17′1.68′′ N, 63°51′2.16′′ W, 440 m, Roraima State), (2) Toototobi (1°45′54.72′′ N 63°37′7.68′′ W, 128 m, Amazonas State), (3) Marari (01°09′39.616′′ N, 64°55′10.145′′ W, 139 m, Amazonas State) in the Yanomami Indian Reserve in the Brazilian Amazon biome, and (4) Salobra (20°12′40″ S, 56°29′30″ W, 117 m, municipality of Miranda, Mato Grosso do Sul State) in the Pantanal biome, Paraguay river basin (Figure 1).

The Yanomami are the major indigenous group living in semi-isolated communities. Each community inhabits ecologically distinct areas with diverse geomorphologic and hydrologic characteristics [18]. Mosquito collections were conducted in the abovementioned communities 1–3 [44,45]. The Parafuri community is in Roraima state, in the Amazonian submontane tropical rainforest ecoregion at 440 m altitude. The Roraima submontane ecoregion is located exclusively inside the Yanomami Reserve at 350–650 m [46]. Mosquito collections were undertaken in 4 Parafuri villages—Komomassipe, Warareu along the Inajá River, and Makabey and Xaruna on the Parima River. Toototobi community is located 170 km south of Parafuri, in a lowland tropical rainforest ecoregion with a maximum of 150 m altitude [46]. Mosquito collections in Toototobi were carried out in 5 villages—Koiobi, Maraxipora, Apiahik, and Raxasi along the Toototobi River, a tributary of the Demini River. Marari community at 139 m altitude is in a lowland Amazonian rainforest area with first to third order rivers and surrounded by high mountains. The mosquito collections were carried out in 4 villages—Taibrapa, Gasolina, Alapusi, and Ahima/Castanha. In Marari, malaria transmission is perennial and periodically intense, and the community is characterized by villages with high population density and high risk of year-round immigration of *Plasmodium*-carriers from highly endemic areas outside the Yanomami territories (Supplementary Table S1).

In the Pantanal biome, collections were carried out in Salobra village, located by the Miranda River. The Pantanal is one of the largest wetlands in the world [47,48] extending over 147,574 km^2^ in Brazil. It is located south of the Brazilian Amazon, with seasonal flooding and dry regimes [47] and low-height shrubs characteristic of savannah-like vegetation (*Cerrado* vegetation). The Pantanal Region extends into areas of Mato Grosso and Mato Grosso do Sul states, as well as parts of Bolivia to the north and Paraguay to the south [47].

### 2.2. Mosquito Collections and Species Identification

The specimens sequenced in this study were field-collected adults and F1-adult progeny. In Parafuri and Toototobi sampling was done bimonthly from January 2013 to July 2014. In the Marari region, collections were done in March 2013 and August 2013, and during the dry season, from September 2014 to March 2015, 4 collections were conducted [44,45]. In Salobra, mosquitoes were collected in September and November 2010, and January, March, May, and July 2011 [27]. Specimens were morphologically identified using the key of Forattini [49] and stored dry individually with silica gel at room temperature for subsequent analysis. The taxonomic nomenclature adopted in this study is that proposed by Foster et al. [11] and defended by Lamas et al. [50].

### 2.3. DNA Extraction

Genomic DNA was extracted from 1 or 2 legs of each anopheline specimen. For each extraction, the legs were macerated in 10 µL of NaCl 0.9%, after which 20 µL of Chelex-100 5% was added. The solution was vortexed, incubated at 99 °C for 10 min, and then centrifuged at 13,000 rpm for 5 min at 25 °C. The supernatant was recovered, and an aliquot was frozen at −20 °C for PCR amplification of the target gene region. The remnant of the mosquito’s body and DNA were deposited and stored at −70 °C, respectively, in the entomological collection of the Faculdade de Saúde Pública (FSP), São Paulo, Brazil.

### 2.4. DNA Amplification

Primers LCO1490 5′-GGTCAACAAATCATAAAGATATTGG-3′ and HCO2198 5′-TAAACTTCAGGGTGACCAAAAAATCA-3′ were used to amplify the barcode region of the *COI* gene, employing the protocol of Bourke et al. [13]. Each reaction was performed with a final volume of 25 µL containing 1× PCR Buffer (Invitrogen), 1.5 mM MgCl_2_ (Invitrogen), 0.2 mM each dNTPs (Amresco), 0.1 μM each primer, 0.625 U Taq *Platinum* polymerase (Invitrogen), 2 µL of DNA, and the remaining volume of ultra-pure water. The thermocycler conditions consisted of 94 °C for 3 min, 5 cycles of 94 °C for 30 s, 45 °C for 90 s, 68 °C for 60 s, followed by 35 cycles of 94 °C for 30 s, 51 °C for 30 s, 68 °C for 60 s, and a final extension at 68 °C for 10 min. PCR products were purified by polyethyleneglycol (PEG) precipitation. For this purification, equal volumes of PCR product and PEG (20% polyethyleneglycol 8000/2.5 M NaCl) were homogenized and incubated at 37 °C for 15 min. Then, it was centrifuged at 13,000 rpm for 15 min at 25 °C and the supernatant was removed. To the pellet were added 200 µL of 70% cold ethanol and then centrifuged at 13,000 rpm for 15 min at 4 °C. The supernatant was discarded, and the wash was repeated. To the dry pellet, 10 µL of ultra-pure water were added.

### 2.5. Sequencing and Alignment

Sequencing was performed in both directions using a Big Dye Terminator cycle sequencing kit v3.1 (Applied Biosystems, Foster City, CA, USA) and the same set of PCR primers. The sequencing products were purified using Sephadex G50 columns (GE Healthcare, Chicago, IL, USA) and analyzed in an Applied Biosystems 3130 DNA Analyzer (PE Applied Biosystems). Sequences were edited in Sequencher v.4.9 software (Genes Codes Corporation, Ann Arbor, MI, USA) and the primer regions removed. Sequences from GenBank or those previously processed in the Systematics Molecular Laboratory (FSP) were also included to serve as references in the analyses. The *COI* gene sequences were aligned by nucleotide using the muscle algorithm [51], implemented in SeaView [52], and then by amino acid using TranslatorX [53].

### 2.6. Species Delimitation

Three methods were employed for species delimitation analysis: Assemble Species by Automatic Partitioning—ASAP [40]; multi-rate Poisson tree processes—mPTP [54]; Refined Single Linkage—RESL [38]. ASAP is a method of species delimitation that uses pairwise genetic distances and ascending hierarchical clustering to build a list of best partitions. The partitions are ranked by score, which is a combination of two metrics: probability of panmixia and the barcode gap width. This method does not require any *a priori* knowledge of number of species, the species composition, or any biological information, such as a phylogeny or intraspecific distances. Multi-rate Poisson tree processes (mPTP) is a phylogeny-aware method that uses differences in mutation rate in a phylogenetic tree to resolve interspecific and intraspecific diversity. It does not rely on *a priori* distance thresholds but requires a bifurcating, non-ultra-metric phylogenetic tree for input. A maximum likelihood (ML) tree was generated with RAxML-HPC BlackBox 8.2.10 [55] on the CIPRES Science Gateway [56], using gene partitioning by codon under the model GTR + G, bootstrapping halted automatically using MRE-based, boot-stopping criterion. A minimum branch length value for the RAxML best tree was calculated (--min_br) to control for very similar sequences and over splitting. This branch length was used as input for the MCMC delimitation analysis. To assess convergence, four independent MCMC analyses were performed. Convergence was reached at 100 million steps. The average standard deviation of delimitation support values among MCMC runs was <0.00005 and they each provided >0.95 support for the ML delimitation. RESL, which is available within the BOLD workbench (www.boldsystems.org, accessed on 24 August 2021), uses p-distances, an intraspecific threshold of 2.2% and single linkage clustering to create groups that are then refined using Markov Clustering and the Silhouette Criterion.

## 3. Results

### 3.1. Species Identification

A total of 763 sequences (216 new sequences—199 from Yanomami and 17 from Pantanal collections, and 547 reference sequences) from *Anopheles*, *Kerteszia,* and *Nyssorhynchus* and *Chagasia* (outgroup) genera were used in mPTP, ASAP, and RESL analyses (Supplementary Tables S2 and S3).

**Figure 2:**
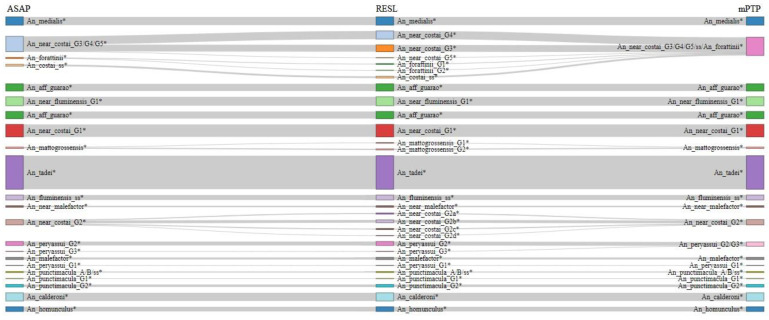
Sankey diagram of the three analyses for the genus *Anopheles*.

**Figure 3:**
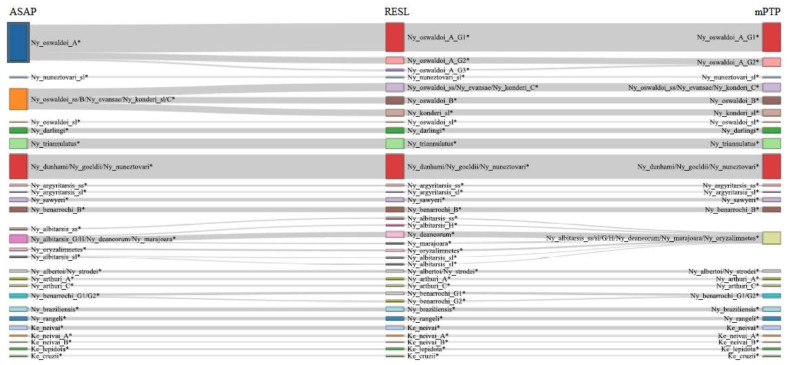
Sankey diagram of the three analyses for the *Nyssorhynchus* and *Kerteszia* genera.

With the exclusion of the outgroup, the mPTP ML delimitation of collected and reference specimens found 45 *COI* clusters, while the ASAP analysis delimited 48 (Figure 2 and Figure 3, Supplementary Table S4). RESL analysis resulted in 63 clusters, with 5 showing maximum intraspecific variation close to or greater than 3% (2.9–3.9%) (Figure 2 and Figure 3, Supplementary Table S5).

### 3.2. Genus Anopheles

Sequences of specimens of *Anopheles* species collected the Yanomami and Pantanal areas showed few differences in the delimitation of the clusters across analyses (Supplementary Table S4). *Anopheles costai* was split into two clusters. The first cluster, *An*. near *costai* G1, was found in Parafuri (RR) and clustered with sequences from Colombia (GenBank accession: JX205128, KF698865), Acre (Brazil), and Amazonas (Brazil). Sequences of the second cluster, *An*. near *costai* G4, were generated from specimens collected in Toototobi and Marari (AM) and were clustered with reference sequences from Acre, Amazonas, and Colombia (GenBank accession: HM022403, HM022404, JX205127) in RESL analysis. *Anopheles* near *costai* G4 clustered with reference sequences from Rondônia in ASAP analysis, and with *Anopheles forattinii* in mPTP analysis. Both clusters displayed a maximum intraspecific variation of 2.6% in RESL analysis (Supplementary Table S5).

*Anopheles fluminensis s.l*. sequences from Parafuri did not cluster with reference sequences of *An. fluminensis s.s*. (GenBank accession: MF381677, MF381699) but with samples from Acre (GenBank accession: MH844300-MH844310) and were named *An*. near *fluminensis* G1.

In the RESL analysis, *Anopheles mattogrossensis* did not cluster with reference sequences as in the mPTP and ASAP analyses. *Anopheles medialis* (new name for *An. intermedius*) and *Nyssorhynchus tadei* (formerly known as *Ny. konderi* B) each clustered separately across all species delimitation analyses. Two clusters that were delimited across analyses could not be identified with the inclusion of reference sequences and are herein named *Anopheles* aff. *guarao* because of morphological similarities with both *Anopheles guarao* and *Anopheles shannoni*.

### 3.3. Genus Nyssorhynchus

Delimitation of the *Nyssorhynchus* sequences from the Yanomami and Pantanal areas showed notable differences across analyses (Supplementary Table S4). *Nyssorhynchus goeldii*, *Ny. nuneztovari*, and *Ny. dunhami* were clustered together and wholly unresolved across analyses. This was also the case for *Ny. konderi* C, *Ny. evansae*, and *Ny. oswaldoi s.s.* The maximum intraspecific variations of the first and second cluster were 3.91% and 1.69%, respectively (Supplementary Table S5).

Although ASAP analysis identified *Ny. oswaldoi* A as a single cluster, mPTP and RESL analyses partitioned *Ny. oswaldoi* A into two clusters. In the mPTP and RESL analyses, *Nyssorhynchus konderi s.l.* was split in two clusters. *Nyssorhynchus konderi* C clustered with reference sequences of *Ny. oswaldoi s.s.*, *Ny. evansae*, and *Ny. konderi* from Paraná and Rondônia, whereas *Ny. konderi s.l.* was well delimited. *Nyssorhynchus nuneztovari s.l*, *Ny. darlingi,* and *Ny. triannulatus* were all clearly delimited across all analyzes and *Ny. oswaldoi* B was well delimited in RESL and mPTP analyses.

### 3.4. Genus Kerteszia

*Kerteszia sequences* were delimited into a *Kerteszia lepidota* cluster and three *Kerteszia neivai* clusters across analyses (Supplementary Table S4). All three *Ke. neivai* clusters were collected in Parafuri. The first cluster was identified as *Ke. neivai s.s*. while the remaining two clusters are herein denoted as *Kerteszia neivai* A and *Kerteszia neivai* B.

### 3.5. Summary of Species Delimitation for Collection Specimens

With respect to the full dataset (test and reference sequences), the mPTP partition was the most conservative (*n* = 45) while the RESL partition was the least (*n* = 63). Relative to test sequences only, between 18 and 21 clusters were detected (Table 1 and Supplementary Table S4). Of these, only the *Ny. dunhami*/*Ny. goeldi*/*Ny. nuneztovari* and *Ny. triannulatus* clusters showed maximum intraspecific variation greater than 3% (Supplementary Table S5).

## 4. Discussion

Correct species identification is a prerequisite for describing vector distribution and biology and is essential for effective malaria management and vector control. However, the morphological identification of adult female mosquitoes can be impaired by the presence of overlapping characters between species [73]. Under such circumstances, molecular data and the *COI* barcode region are particularly useful for species identification [13,74,75].

The Amazon region harbors numerous species of Anophelinae and some of them are recognized as vectors of *Plasmodium* spp. [13]. This region is also where most of the Brazilian malaria cases occur [76] and the people at greatest risk of infection are those from the indigenous community [77,78]. However, due to the geographical isolation and inaccessibility of the Yanomami territories, little is known about the vectors of *Plasmodium* spp. present in the area [45]. There are also few studies on malaria vectors in the Pantanal region [19], despite being a potentially important region for *Plasmodium* transmission [27]. Our study has therefore sought to expand on the limited knowledge of Anophelinae fauna in Salobra (Pantanal) and in three Yanomami communities (Toototobi, Marari, and Parafuri).

### 4.1. Nyssorhynchus and Kerteszia Genera

The genera *Nyssorhynchus* [4,76,79] and *Kerteszia* [80] include species that are the dominant vectors in the Amazon and Atlantic Forest regions, respectively. Concerning the Yanomami territory, Sánchez-Ribas et al. [45] found *Ny. darlingi* in Parafuri and Marari, but not in the Toototobi locality, suggesting that other species are likely involved in malaria transmission, such as *Ny*. *triannulatus* and *Ny*. *nuneztovari* that have been found infected with *Plasmodium* spp. in areas throughout the Amazon River basin [4,79].

Analyses of *COI* and ITS2 sequences have previously supported the separation of at least five species in the Oswaldoi–Konderi Complex: *Nyssorhynchus oswaldoi s.s.*, *Ny. oswaldoi* A, *Ny. oswaldoi* B, and at least two of *Ny. konderi* (*Ny. konderi* of Sallum and *Ny.* near *konderi* or *Ny. konderi* B) [34,35,81], after *Ny. konderi* was removed from synonymy with *Ny. oswaldoi* [82]. *Nyssorhynchus konderi* is susceptible to *Plasmodium* infection [83], but its epidemiological importance is still unknown. Based on morphological and molecular data, Saraiva and Scarpassa [84] identified the group informally denoted *Ny.* near *konderi* [35] or *Ny*. *konderi* B [34] as the new species, *Nyssorhynchus tadei* (named *Anopheles* (*Nyssorhynchus*) *tadei*).

This study reveals two *COI* clusters, or putative species, of *Ny. konderi* and *Ny. tadei*in Salobra. The first cluster is formed by “*Ny. konderi* of Sallum” [35] and here denoted *Ny. konderi s.l.* Specimens of this putative species have previously been recorded in Acre and Amapá (referred to as *Ny*. *konderi* in Bourke et al. [13]). The second cluster includes specimens previously named *Ny. konderi* C and the reference sequences of *Ny. konderi* from Paraná and Rondônia [13]. *Nyssorhynchus oswaldoi s.s.* and *Ny. evansae* sequences clustered with *Ny. konderi* C. The observed clustering can be explained by the lack of phylogenetic signal in the *COI* barcode fragment to separate the species that evolved recently. *Nyssorhynchus tadei* was clustered with specimens from Acre, Amazonas, and Ecuador, and previously referred to as *Ny*. near *konderi* in Ruiz-Lopez et al. [35] and Bourke et al. [13].

*Nyssorhynchus oswaldoi s.s.* has been found in the states of São Paulo, Espírito Santo [29], Rio de Janeiro, Acre, and Amazonas (in the city of Coari) [35] and is herein registered in the municipality of Salobra, Pantanal, state of Mato Grosso do Sul. This species is not involved in the transmission of *Plasmodium* spp. [29,67]. A closely related species, *Ny*. *oswaldoi* B, has a large geographical distribution, occurring in Putumayo, Antioquia, Caquetá, and Norte Santander in Colombia; Ocama, Venezuela; Province Orellana, Ecuador; Amapá, Brazil; and Saint Andrew/Saint David Island, Trinidad/Tobago. Results of this study showed that the species also occurs in the region of the Toototobi community. Females of *Ny*. *oswaldoi* B were found naturally infected with *Plasmodium* spp. in Putumayo, Colombia [68], indicating the potential of the species involvement in *Plasmodium* spp. transmission across its distribution area. In addition, Ruiz-Lopez et al. [35] pointed out that *Ny*. *oswaldoi* B could be *Ny*. *aquacaelestis*, a species that was described based on specimens collected in Panama and is currently the synonymy of *Ny*. *oswaldoi s.s*. Further research will be necessary to define whether *Ny*. *oswaldoi* B is an undescribed species or *Ny*. *aquacaelestis*. In the Toototobi and Parafuri communities, we also registered the presence of *Ny. oswaldoi* A. This species has been found in areas across the Amazon River basin, in the states of Acre, Amazonas, Mato Grosso, Pará, and Rondônia in Brazil, and in the department of Amazonas, Colombia [35,85,86,87]. *Nyssorhynchus oswaldoi* A is provisionally involved in malaria transmission in Acre, Brazil, because it was the only species registered in Acre where females were found naturally infected with *Plasmodium* [72]. The mPTP analysis splits *Ny. oswaldoi* A into two clusters, which are separated by a minimum K2P distance of 2.16%. Although this split could indicate a potential new species within *Ny. oswaldoi* A, this hypothesis is not supported by the other analyses employed in the study. The potential of both *Ny. oswaldoi* B and *Ny. oswaldoi* A involvement in the malaria transmission across their geographical distribution needs further investigation.

The Nuneztovari Complex comprises *Nyssorhynchus goeldii*, *Nyssorhynchus nuneztovari*, *Nyssorhynchus nuneztovari* A, and *Nyssorhynchus dunhami* [34]. *Nyssorhynchus nuneztovari s.s.* is considered a primary vector in Venezuela and Colombia [64,65]. Mosquito females identified as *Ny*. *nuneztovari* were found infected with *Plasmodium* parasites and considered a vector in modified forest in the Brazilian Amazon [88]. *Nyssorhynchus nuneztovari* A is a local vector in the Brazilian Amazon [58,69,70,71]. *Nyssorhynchus dunhami* has been identified in several regions of the Brazilian Amazon [71] and was found naturally infected in the peridomestic environment in Iquitos, Peru [63]. The role of these species as vectors in the areas studied is unknown. The phylogenetic resolution of *Ny*. *goeldii*, *Ny.nuneztovari*, and *Ny. dunhami* has previously proved difficult at the *COI* locus [13]; however, Foster et al. [34] showed an unambiguous separation between *Ny. dunhami* and the cluster composed of *Ny. goeldii* and *Ny. nuneztovari* using concatenated sequence data of the single copy nuclear *white* and *CAD* genes, and the *COI* barcode region. Two specimens, from Marari and Parafuri, that did not group with Nuneztovari Complex cluster, are here denoted *Ny. nuneztovari s.l.*

Species of the genus *Kerteszia* occupy habitats with abundant forest bromeliads and high rainfall [89] and are associated with the so-called human bromeliad malaria and zoonotic malaria [12,19]. In Brazil, the distribution of *Kerteszia* species is mainly in areas of Serra do Mar in the Atlantic rainforest, although there are records of *Kerteszia cruzii* in Parque do Iguaçu [90] and *Ke. neivai* in Jaú National Park in the state of Amazonas [91]. In the present study, the *Ke. neivai* specimens collected in Parafuri were split into three clusters and denoted *Ke. neivai s.s.*, *Ke. neivai* A, and *Ke. neivai* B. *Kerteszia neivai s.s.* is prevalent on the Colombian Pacific coast, where it transmits *Plasmodium* spp. [61]. Although it was once found infected with sporozoites by a non-identified *Plasmodium* spp. in the state of Amazonas [92], its role as vector of human malaria in the Amazon is not yet known. *Kerteszia lepidota* was also collected in Parafuri. Despite having been implicated previously as a malaria vector in Colombia [60], subsequent studies found that *Kerteszia lepidota* was misidentified and the local vector was *Ke. pholidota* [93,94]. *Kerteszia lepidota* is therefore not considered to be an important vector in the Yanomami regions studied herein.

### 4.2. Anopheles Genus

*Anopheles costai* is morphologically similar to both *Anopheles forattinii* and *Anopheles mediopunctatus* based on females characteristics [95,96], but these species can be easily identified by the male genitalia, pupa, fourth instar larva, and scanning electron microscopy of eggs. Sequence data can help to separate females of these species and uncover species that remain undescribed, especially in remote areas that have been barely sampled, such as the Yanomami region and other areas of the Brazilian Amazon. Recently, Bourke et al. [13] showed that *An. costai* is a highly diverse species complex with three to six phylogenetic lineages. In this study, the ASAP analysis resolves up to four clusters (*An. costai s.s.*, *An.* near *costai* G1, *An.* near *costai* G2, and *An.* near *costai* G2/G4/G5) in the full dataset (newly sequenced and reference sequences). In the localities sampled only *An.* near *costai* G1 and *An.* near *costai* G4 were found. Having been previously collected in the states of Acre and Amazonas in Brazil and in Colombia [13], these putative species are herein recorded in Parafuri, in the state of Roraima, and Toototobi and Marari, in the state of Amazonas, respectively.

The type-locality of *Anopheles fluminensis* is the municipality of Itaperuna (Rio de Janeiro state). The species was registered in the states of São Paulo [97] and Paraná [98]. Neves et al. [99] found this species positive for *Plasmodium malariae* in an indigenous village in the Vale do Rio Branco, Itanhaém, São Paulo. Bourke et al. [13] detected two clusters of *An. fluminensis* in collections from rural settlements in Acre, the first denoted *An. fluminensis s.s.* and the second *An.* near *fluminensis* G1. The present study identified the same two clusters. Because the *An.* near *fluminensis* G1 cluster displays a maximum intraspecific variation close to 3%, further investigation will be necessary to verify whether *An.* near *fluminensis* G1 represents cryptic species. *Anopheles medialis* and *An. mattogrossensis* are widely distributed in Brazil [45,100]. Despite the register of *Plasmodium* infection in *An. mattogrossensis* [57] and *An. medialis* (as *An*. *intermedius*) [58,59], the role of these species in *Plasmodium* transmission is poorly known in Brazil. Both species were found in the areas studied in the Yanomami territories. Further investigations will be necessary to verify whether these species are involved in the *Plasmodium* transmission in this region.

## 5. Conclusions

This study increases the scant knowledge of Anopheline species in the Yanomami and Pantanal regions. At least 18 species were found in these regions. Our findings provide an important basis for further studies that seek to explore the relative roles of Anophelinae species in *Plasmodium* transmission among vulnerable indigenous communities in the Amazon. Additionally, a baseline for mosquito and vector diversity in the Pantanal is provided, where extant data are lacking, and a few malaria cases occurs yearly.

## Figures and Tables

**Table 1:** Species and putative species data collected in Yanomami and Pantanal areas.

Species	Status *Plasmodium* Vector [Reference]	References for Species Found in This Study
*An. mattogrossensis*	Its role as *Plasmodium* vector is poorly known in Brazil [57]	
*An. medialis*	Its role as *Plasmodium* vector is poorly known in Brazil [58,59]	
*Ke. lepidota*	It is a *Plasmodium* vector in Colombia [60]	
*Ke. neivai*	It is a *Plasmodium* vector in localities on Pacific Coast, Colombia [61]	
*Ny. darlingi*	It is the dominant *Plasmodium* vector in several regions of the Brazilian Amazon [62]	
*Ny. dunhami*	It has been found naturally *Plasmodium* infected in Iquitos, Peru [63]	
*Ny. goeldii*	It has been found naturally *Plasmodium* infected in locations across the Brazilian Amazon [62]	
*Ny. nuneztovari*	It is a primary *Plasmodium* vector in Venezuela and Colombia [64,65]	
*Ny. evansae*	Unknown [66]	
*Ny. oswaldoi s.s.*	Unknown [29,67]	
*Ny. oswaldoi* B	Potential *Plasmodium* vector in Putumayo, Colombia [68]	
*Ny. konderi* C	Unknown	[13]
*Ny. konderi s.l.*	Unknown	[13,35]
*Ny. nuneztovari s.l.*	*Nyssorhynchus nuneztovari* A is a local *Plasmodium* vector in localities across the Brazilian Amazon [58,69,70,71]	
*Ny. oswaldoi* A	Potential *Plasmodium* vector in Acre [72]	
*Ny. tadei*	Local vector of *Plasmodium* across the Brazilian Amazon [4]	
*Ny. triannulatus*	It was found naturally *Plasmodium* infected in localities across the Brazilian Amazon [58,62]	
*An.* aff. *guarao*	Unknown	-
*An.* aff. *guarao*	Unknown	-
*An.* near *costai* G1	Unknown	[13]
*An.* near *costai* G4	Unknown	[13]
*An.* near *fluminensis* G1	Unknown	[13]
*Ke. neivai* A	Unknown	-
*Ke. neivai* B	Unknown	-

## Data Availability

*COI* sequences of samples collected in Yanomami and Salobra areas have been deposited in the GenBank database (https://www.ncbi.nlm.nih.gov/genbank/, accessed on 23 April 2021) (GenBank accession: MZ014105–MZ014320).

## Supplementary Materials

The following are available online at https://www.mdpi.com/article/10.3390/genes12121995/s1, Table S1: Information on distances, travel modalities, and times (during the dry and rainy seasons) for the Yanomami Indian communities Tootootobi, Parafuri, and Marari and villages were included in this study, 2014–2015, in Brazil, Table S2: Sample information of collection specimens and GenBank accession number, Table S3: Reference sequences information, Table S4: Species groups resulting from the ASAP, mPTP, and RESL analyses, Table S5: Cluster sequences from RESL analysis.
