# Supplementary material for: Molecular Analysis Reveals a High Diversity of Anopheline Mosquitoes in Yanomami Lands and the Pantanal Region of Brazil
Source: Genes (Basel). 2021 Dec 16;12(12):1995. doi: 10.3390/genes12121995 (PMC8701885; doi:10.3390/genes12121995)
Supplement: Supplementary file 1 [file genes-12-01995-s001.zip › Table_S1.pdf]

Molecular analysis reveals a high diversity of anopheline mosquitoes in Yanomami lands and the Pantanal Region of Brazil

Teresa Fernandes Silva-do-Nascimento[1], Jordi Sánchez-Ribas[1,2,3], Tatiane M. P. Oliveira[4*], Brian Patrick Bourke[5,6], Joseli Oliveira-Ferreira[1], Maria Goreti Rosa-Freitas[1,7], Ricardo Lourenço de Oliveira[1], Mariana Marinho-e-Silva[1,8], Maycon Sebastião Alberto Santos Neves[1], Jan E. Conn[9,10#] and Maria Anice Mureb Sallum[4#]

[1] Laboratório de Mosquitos Transmissores de Hematozoários, Instituto Oswaldo Cruz Fundação Oswaldo Cruz, Rio de Janeiro, RJ, Brazil.
[2] Laboratório de Imunoparasitologia. Instituto Oswaldo Cruz, Fundação Oswaldo Cruz, Rio de Janeiro, RJ, Brasil.
[3] Distrito Sanitário Especial Indígena Yanomami, Roraima, Brazil.
[4] Departamento de Epidemiologia, Faculdade de Saúde Pública, Universidade de São Paulo, São Paulo, SP, Brasil.
[5] Walter Reed Biosystematics Unit, Museum Support Center MRC-534, Smithsonian Institution, 4210 Silver Hill Rd. Suitland MD, 20746, USA.
[6] Department of Entomology, Smithsonian Institution – National Museum of Natural History, 10th St NE & Constitution Ave NE, Washington, DC, 20002, USA.
[7] Geniac Ltda, São Paulo, Brasil.
[8] Instituto Nacional da Propriedade Industrial, Rio de Janeiro, RJ, Brasil.
[9] Wadsworth Center, New York State Department of Health, Albany, NY, 12159 USA.
[10] Department of Biomedical Sciences, School of Public Health, State University of New York, Albany, NY,12222 USA.

Table S1 - Information on distances, travel modalities and times (during the dry and rainy seasons) for the Yanomami Indian communities Tootootobi, Parafuri and Marari and villages were included in this study, 2014-2015, in Brazil.

**Communities/ villages**	**Distance from health center (km)**	**Transport- Boat (h)**	**On foot (h)**	**Transport- Boat (h)**	**On foot (h)**
		Dry season	Dry season	Rainy season	Rainy season

Communities/ villages	Distance from health center (km)	Transport- Boat (h)	On foot (h)	Transport- Boat (h)	On foot (h)
**Tootootobi/**					
Mahaxipoha	14.9	6 h	7 h	4 h	-
Raxasi	5.2	1 h	1 h30 min	40 min	-
Apiahik	6.4	3 h	2 h	2 h	-
Koiobi	8.1	0	1 h40 min	-	2 h
**Parafuri/**					
Xaruna	7.3	4 h	-	4 h	-
Makabey	15.2	3 h30 min	-	3 h 30 min	-
Warareu	4.9	40 min	-	40 min	-
Komomassipe	7.4	1 h40 min	-	1 h40 min	-
**Marari/**					
Alapusi	5.3	-	1 h40 min	-	2 h
Castanha/Ahima*	0	0	0	0	0
Taibrapa	2.2	0	20 min	-	25 min
Gasolina	5		1 h45 min	-	2 h15 min
Taibrapa	15.9	6 h	-	4 h	-

* By the headquarters of the Health Unit.
